# The Classification of Aneurisms

**Published:** 1895-07-06

**Authors:** A. Pearce Gould

**Affiliations:** Senior Assistant Surgeon to the Middlesex Hospital


### The Classification of Aneurisms.

By A. Pearce Gould, F.R.C.S., Senior Assistant Surgeon to the Middlesex Hospital.

In a previous article we have seen the large part that injury plays in causing aneurism. A clear distinction must be drawn between those cases in which injury causes disease of the wall of an artery which ultimately develops into an aneurism, or cases in which slight injury is the final exciting cause of the yielding of a previously diseased artery, and the cases where injury is the sole, and the direct and immediate cause of aneurism. Where an aneurism arises independently of injury, or occurs as the sequel of disease in the arterial tissues, it is called an idiopathic aneurism, or a pathological aneurism. But where an aneurism is the immediate and direct result of an injury to the artery, and is quite independent of any previous disease of the vessel, it is a traumatic aneurism. This distinction is not unimportant, for in cases of idiopathic aneurism there is always more or less disease of the arteries generally, and sometimes to an extreme degree, while in cases of traumatic aneurism there may be, and often is, no disease of the vascular system at all, and the conditions are thereby more favourable for obtaining a wholly satisfactory result from treatment. It is a purely local affection, while in the other cases the local tumour is an exaggeration, an advanced stage, of a general disease. What we wrote in the previous article on the classification of aneurisms applied entirely to the idiopathic variety. Traumatic aneurisms are divided into two great groups—circumscribed and diffused. By a "circumscribed traumatic aneurism" is meant a tumour which possesses a limiting sac, and by a "diffused traumatic aneurism" one that has no limiting sac. A diffused traumatic aneurism is like a ruptured idiopathic aneurism in its effects, but inasmuch as it is usually diffused from first to last, and the diffusion of the blood is not due to the rupture of a sac, it cannot rightly be called a ruptured aneurism. Probably the best name for such a case is "ruptured artery." Another very important group of aneurisms is that in which there is an abnormal direct communication between an artery and a vein—arterio-venous aneurism, and of these there are two distinct varieties. One consists of a direct communication between an artery and a vein, the opening in one vessel being adherent to the opening in the other. In such a case the blood is forcibly propelled from the artery into the vein and distends that vessel into a varix, but the varix pulsates and has other characters like an aneurism, and so the affection is known as "Aneurismal Varix." In the other form there is a distinct aneurismal tumour connected with an artery, and a communication between its sac and a vein. Blood passes from the aneurism into the vein, and the vessel becomes dilated and varicose, but the important feature of the case is the existence of the aneurism. These cases are designated "Varicose Aneurisms."

These cases are most often the direct result of a wound, but in exceptional instances they arise independently of injury, as, for instance, when a large aneurism of the aorta, as a result of pressure, opens into the pulmonary artery or the innominate vein. They must therefore be placed in a group by themselves, and not as a sub-variety of traumatic aneurisms. The last important variety of aneurism is that formed of a single enlarged tortuous vessel, or of a number of such vessels freely inter-communicating. These tumours are really a form of angeioma, but they are very commonly designated "Aneurism by Anastomosis."

We may, therefore, draw up the following table of aneurisms:—

| | |
|---|---|
| Fusiform or Tubular Aneurism. | |
| Mixed Aneurism. | |
| Sacculated Aneurism | Circumscribed, Leaking, Ruptured. |
| Traumatic Aneurism | Circumscribed, Diffused or Ruptured Artery. |
| Anterio-venous Aneurism | Aneurismal Varix, Varicose Aneurism. |
| Aneurism by Anastomosis. | |

*The Aneurismal Sac.*—In fusiform and tubular aneurism the wall or sac of the tumour contains all three coats of the artery. The inner coat is the seat of more or less advanced atheromatous change, and calcareous plates sometimes of great size are often met with, proving in the most evident manner the presence of the tunica interna. The middle coat is thinned, the muscular bundles being more or less widely separated, and often the seat of fatty change. The outer coat is in most cases thickened by the addition of new fibrous tissue. It is in cases of sacculated aneurism that the structure of the sac presents chief interest. In certain rare cases the sac consists of all three coats of the artery; this only occurs where the aneurism is quite small, and has been formed by the yielding of large segment of the arterial wall. In fact, the more nearly a sacculated aneurism approaches in nature to a fusiform aneurism the more probably will it contain in its sac all the coats of the artery. It must be clearly understood that these cases are quite exceptional. As a rule the inner coat of the artery is lost at the mouth of the aneurism, and no trace of it is found in the sac, and the same is true of the middle coat, save that a few scattered bundles of muscle-fibre may be found in the sac of small aneurisms. It is this fact which explains the immense gravity of a sacculated aneurism and its great liability to growth and rupture. This thin sac is strengthened in two or three ways. The pressure of the tumour upon the tissue around it sets up a low form of plastic "inflammation," with formation of fibrous tissue which thickens the sac upon its outer side. As the tumour enlarges it displaces the surrounding structures, and these by their tension strengthen and support the sac, and add to its thickness and power of resistance. Clots also form within the sac, but when lining its walls serve to strengthen it. When they adhere to the sac, they become organised into fibrous tissue, and then they form a great addition to the thickness and strength of the sac. Of these clots we must say more when we come to deal with the process of cure of an aneurism.

